# The *Burkholderia pseudomallei* intracellular ‘TRANSITome’

**DOI:** 10.1038/s41467-021-22169-1

**Published:** 2021-03-26

**Authors:** Yun Heacock-Kang, Ian A. McMillan, Michael H. Norris, Zhenxin Sun, Jan Zarzycki-Siek, Andrew P. Bluhm, Darlene Cabanas, Robert E. Norton, Natkunam Ketheesan, Jeff F. Miller, Herbert P. Schweizer, Tung T. Hoang

**Affiliations:** 1 School of Life Sciences, University of Hawaiʻi at Mānoa, Honolulu, HI USA; 2 Townsville Hospital, Townsville, QLD Australia; 3 Faculty of Medicine, University of Queensland, Brisbane, Australia; 4 Science and Technology, University of New England, New South Wales, Australia; 5 Department of Microbiology, Immunology, and Molecular Genetics, and the California NanoSystems Institute, University of California, Los Angeles, CA USA; 6 Pathogen and Microbiome Institute, Northern Arizona University, Flagstaff, AZ USA; 7 Present Address: Department of Geography and Emerging Pathogens Institute, University of Florida, Gainesville, FL USA

**Keywords:** Model prokaryotes, Bacteriology, Pathogens, Transcription, Infection

## Abstract

Prokaryotic cell transcriptomics has been limited to mixed or sub-population dynamics and individual cells within heterogeneous populations, which has hampered further understanding of spatiotemporal and stage-specific processes of prokaryotic cells within complex environments. Here we develop a ‘TRANSITomic’ approach to profile transcriptomes of single *Burkholderia pseudomallei* cells as they transit through host cell infection at defined stages, yielding pathophysiological insights. We find that *B. pseudomallei* transits through host cells during infection in three observable stages: vacuole entry; cytoplasmic escape and replication; and membrane protrusion, promoting cell-to-cell spread. The *B. pseudomallei* ‘TRANSITome’ reveals dynamic gene-expression flux during transit in host cells and identifies genes that are required for pathogenesis. We find several hypothetical proteins and assign them to virulence mechanisms, including attachment, cytoskeletal modulation, and autophagy evasion. The *B. pseudomallei* ‘TRANSITome’ provides prokaryotic single-cell transcriptomics information enabling high-resolution understanding of host-pathogen interactions.

## Abstract (web summary)

Prokaryotic cell transcriptomics has been limited to mixed or sub-population dynamics and individual cells within heterogeneous populations. Here the authors develop a ‘TRANSITomic’ approach to profile transcriptomes of single *Burkholderia pseudomallei* cells as they transit through host cell infection.

## Introduction

Prokaryotic cells undergo drastic global gene-expression changes when they encounter varying spatiotemporal niches. Such changes cannot be observed by transcriptomic analysis of mixed populations at a fixed temporal or spatial niche. We previously presented numerous potential applications for prokaryotic single-cell transcriptomics (ref. ^1^, Supplementary Fig. 1)^1^. Here we employ single-cell transcriptomic analysis to understand the ‘TRANSITome’ of *Burkholderia pseudomallei* (*Bp*), discovering pathophysiological processes during host cell infection.

Melioidosis, first described in 1912^2^, is an emerging tropical disease that is a significant threat to human health, caused by the facultative intracellular pathogen *Burkholderia pseudomallei* (*Bp*)^3^. Although the prevalence of *Bp* and melioidosis is expanding globally due to increasing awareness by clinicians and researchers^4–14^, there are still 165,000 predicted annual cases with an estimated mortality rate of 54%^15^. *Bp* can infect most tissues in the human body including bone, joint, skin, lung, liver, spleen, central nervous system (CNS), and genitourinary tract leading to diverse clinical manifestations, ranging from localized acute abscesses, bacteremia, septic shock, chronic infections, and, in rare cases, CNS infections, including brainstem encephalitis, making diagnosis difficult^3,16–19^. To establish infection in a wide range of cell types, *Bp* must possess a complex network of virulence factors/pathways to survive in these different environments. The *Bp* genome contains two chromosomes, 4.07 and 3.17 megabase pairs each, that control basic metabolic pathways and accessory functions, respectively^20^. Thus far, only a fraction of the complex genome is understood in terms of *Bp* successfully establishing an intracellular niche.

There are a number of known virulence factors that have been characterized in *Bp*, including capsule^21^, lipopolysaccaride^22^, type III and VI secretion systems^23–28^, and BimA^29,30^. During its intracellular lifecycle, *Bp* attaches to host cells and gets internalized by phagocytosis or an unknown mechanism^31^, followed by vesicular escape using the *Burkholderia* secretion apparatus, a type III secretion system (T3SS_Bsa_), to gain entry to the host cell cytoplasm^23,25,26^. *Bp* uses BimA, which functions through molecular mimicry as an Ena/VASP analog, to polymerize host cell actin^30,32^ and its secondary flagella locus^26^ to move freely within the host cell cytoplasm. Spread to neighboring cells is then achieved by protruding and fusing host cell membranes with the virulence-associated type VI secretion system leading to the formation of a multinucleated giant cell (MNGC)^24,26–28^. Although much of the *Bp* intracellular lifecycle has been elucidated, a large number of hypothetical/putative proteins lack characterization^33^, suggesting a major deficiency in the current working knowledge of *Bp* pathogenesis and physiology.

In this work, we use single prokaryotic cell transcriptomics to enhance the current understanding of the complex transcriptional landscape of *Bp* during host cell infection. Due to the intricate nature of *Bp* intracellular pathogenesis, we explore the transcriptomic profile of *Bp* in three distinct stages of host cell transit, the vacuole, cytoplasm, and membrane protrusion to better define this complex process and identify hypothetical proteins critical for this process. This investigation of the *Bp* TRANSITome identifies and assigns virulence functions to several hypothetical proteins important for host cell infection.

## Results and discussion

### *Bp* gene expression flux in host cells

**Fig. 1:**
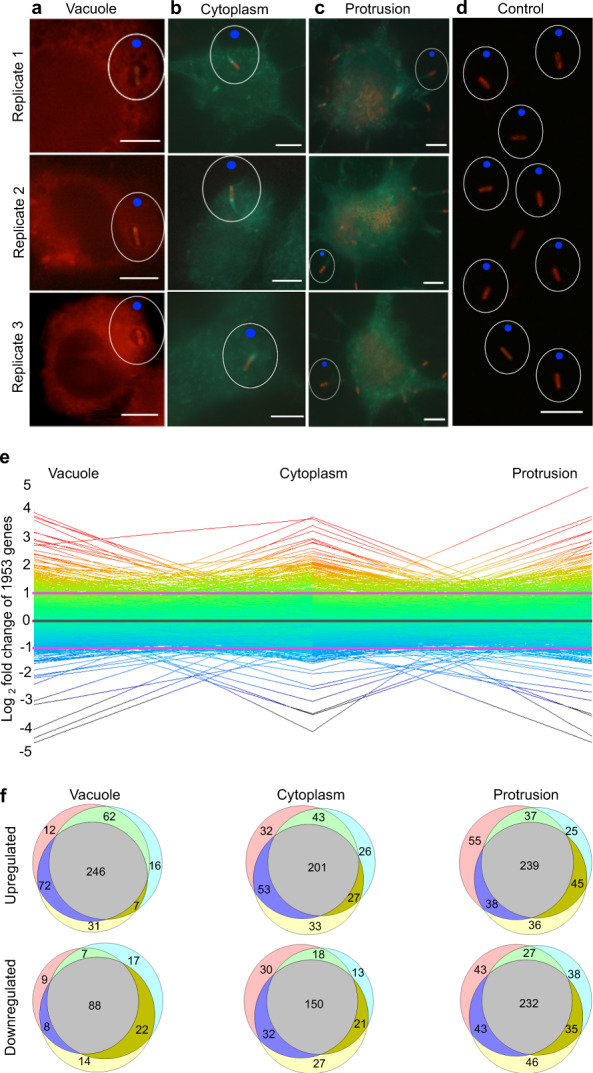
Single *Bp* cell isolation and the TRANSITome. **a**–**d** YFP-RFP-tagged *Bp* infected RAW264.7 cells were fixed and stained red (plasma and vacuolar membranes) and green (actin). Single *Bp* cells were isolated from macrophage vacuoles (**a**), cytoplasm (**b**), and during membrane protrusion (**c**). *Bp* control cells were grown in DMEM medium and isolated as the baseline for gene expression analysis (**d**). Scale bars = 5 μm. Single bacterial cells were cut with the focused laser (white circle) and catapulted using the low-intensity unfocused laser (blue dot) by LCM. **e** *Bp* undergoes dynamic gene expression changes relative to the control cells as it transits through the host cell. Each line represents a single gene with log_2_FC represented in a rainbow color scheme (red lines are upregulated, black lines are downregulated). **f** Genes upregulated (log_2_FC > 1) and downregulated (log_2_FC <−1) showed high reproducibility among biological triplicates. Each color circle represents a biological replicate with gray representing overlap in all biological replicates. Numbers represent the number of genes within each overlapping section of the Venn diagram.

*Bp* transiting through the host experiences various environmental niches, starting from host cell entry into an intracellular vacuole, escaping from the vacuole into the host cell cytoplasm, and finally protruding toward neighboring host cells, spreading the infection^26^. Therefore, we hypothesized that, as *Bp* transits through its intracellular lifecycle, gene-expression is altered to accommodate each unique environmental niche. To probe this hypothesis, we employed our recent method of using laser capture microdissection (LCM)^34^ and total transcript amplification^35^ to isolate single *Bp* cells at each stage of intracellular infection and determined their transcriptional profiles, hereafter referred to as the *Bp* TRANSITome (Fig. 1a–d, Supplementary Movie 1). Comparing single *Bp* cells isolated from various stages of intracellular infection (Fig. 1a–c) to those grown in vitro (Fig. 1d) via microarray analysis, we show that 1953 genes are differentially expressed in a stage-specific manner (Fig. 1e, Supplementary Dataset 1). Many genes show niche-specific expression, indicating dynamic global control of functions at each stage of infection (Fig. 1e, Supplementary Fig. 1a). Biological triplicates from each stage of infection showed high reproducibility for many genes, supporting the validity of this approach to analyze the gene expression of intracellular pathogens (Fig. 1f). Genes showing high reproducibility likely represent conserved functions while genes showing high variability between biological triplicates could be distinguished as a class of genes to study due to variable expression during intracellular infection. Microarray data were validated via qRT-PCR on three selected stage-specific genes and three housekeeping genes (Supplementary Fig. 2a), exhibiting high correlation to validate this method for the investigation of intracellular pathogens (Supplementary Fig. 2b).

We observed known virulence factors that show specific expression patterns during the three defined stages of *Bp* intracellular infection (Supplementary Fig. 3). For example, *Bp* uses its T3SS_Bsa_ to escape the vacuole to gain entry into the host cell cytoplasm^36^ and this is confirmed by the upregulation of numerous type III secretion system genes during the vacuole stage of infection (Supplementary Fig. 3a). All type VI secretion systems present in the *Bp* genome are differentially expressed compared to *Bp* grown in vitro, suggesting that they may be important for maintenance of intracellular infection beyond the known function of host cell membrane fusion^24,27,28^ (Supplementary Fig. 3b). While only portions of each type VI secretion system show expression in the TRANSITome, including a lack of Hcp1 (BPSS1498) expression, we hypothesize that this is due to the limited temporal resolution. For example, a previous study identified that type VI secretion system one (T6SS-1) was induced by glutathione in the cytoplasm of RAW264.7 murine macrophages^37^. They show that *hcp1* is highly expressed 8 h post infection^37^, a time point excluded from the present study. However, another study does show expression of *BPSS1498* at time points that correlate to our study^38^. The increased expression of *BPSS1498* over the first 6 h of infection^38^ and by glutathione induction^37^ likely represent the general trend of all *Bp* within the intracellular environment. During intracellular infection, each individual *Bp* cell is experiencing a slightly different microenvironment that could explain the lack of *BPSS1498* expression in our dataset compared to the previous studies. These results taken together, could suggest that the expression of these systems is highly dynamic and responsive to microenvironments within the host cell. Other significant pathways including flagella and chemotaxis genes, pilus and fimbriae genes, aerobic and anaerobic energy metabolism genes, phosphate transport genes, and potential virulence factors show significant differential regulation throughout the *Bp* TRANSITome highlighting their potential importance during infection (Supplementary Fig. 3c–g). Although the differential regulation of genes with predicted or known function is significant, it is much more intriguing that many genes of unknown functions are also differentially regulated throughout the *Bp* TRANSITome.

### TRANSITome reveals uncharacterized virulence factors

The *Bp* TRANSITome consists of ~30% of genes annotated as hypothetical or putative, having no assigned function (Supplementary Fig. 1b). We endeavored to investigate these genes and hypothesized that many of the hypothetical genes expressed in the TRANSITome contribute to *Bp* intracellular infection. To investigate the function of these unknown genes in cellular pathogenesis, we targeted 206 genes and successfully created 191 in-frame deletional mutants of genes showing distinct expression patterns during the *Bp* intracellular lifecycle (Supplementary Fig. 4a). To assess their role in intracellular infection, these hypothetical/putative genes were deleted in the naturally competent prototype strain *Bp* 1026b using λ-red recombineering^39^. Of the hypothetical genes that showed stage-specific expression, ~100 genes showed high expression in the vesicular stage of infection, while ~55 genes showed upregulation during cytoplasmic replication, and ~74 genes were highly expressed during the process of spreading by protrusion toward neighboring cells (Supplementary Fig. 4a). The stage-specific expression of these hypothetical proteins suggests that they may contribute to different processes during intracellular infection.

The 191 mutants were screened with a qualitative cell fusion assay in RAW264.7 murine macrophages, identifying 11 mutants that showed reduced cell fusion compared to wild-type *Bp* (Supplementary Fig. 4c, Supplementary Table 1). Seven of the attenuated mutants are in genes upregulated during the vacuole stage of infection, two mutants in genes highly expressed in the cytoplasm, and two highly expressed during the protrusion stage of infection (Supplementary Fig. 4b). Of these 11 genes, four, *BPSL0097*, *BPSL0636*, *BPSL1126*, and *BPSL1422*, have not been identified in the literature to our knowledge while seven have had some reference. BpeT and BpeS, regulators of the RND efflux pump BpeEF-OprC, responsible for co-trimoxizole resistance, also control expression of *BPSL1390*^40^. *BPSL2714* and *BPSS1780* are co-expressed with 165 other genes under similar conditions^41^. In addition, a homolog of BPSS1780 was detected in purified outer membrane fractions from *B. mallei*^42^. *BPSS0015* is expressed in early stationary phase culture of *Bp*, but further characterization of the function of this protein has not been carried out^43^. *BPSS1265* was one of 49 genes deleted during clinical treatment of melioidosis but no characterization of the function of this gene was undertaken^44^. *BPSS1818* was downregulated in a N-acylhomoserine lactone synthase mutant strain of *Bp* indicating that it is potentially tied to quorum sensing regulation^41^. BPSS1860 has been previously identified as part of the *Bp* core secretome suggesting an extracellular function^45^. All references to these genes are peripheral suggesting a more in-depth study should be undertaken. Therefore we decided to take a detailed functional characterization of all 11 genes identified through the *Bp* TRANSITome.

The involvement of these hypothetical proteins during the course of host cell infection was further assessed via a quantitative attachment, invasion, and intracellular replication assays (Supplementary Fig. 4d, Supplementary Table 1). The *BPSL0097* and *BPSS1860* mutants showed drastic decreases in attachment at 5 and 10% of wild type, respectively, while other mutants showed moderate defects in this process (Supplementary Table 1). The *BPSL0636* mutant was the only strain to show a significant defect in invasion with a 75% decrease compared to wild type (Supplementary Table 1). Six mutants, *BPSL0097*, *BPSL1126*, *BPSL1390*, *BPSL1422*, *BPSL2714*, and *BPSS0015*, showed defects in intracellular replication between 2 and 6 h post infection, ranging from 17 to 75% wild-type replication while eight mutants, *BPSL0097*, *BPSL0636*, *BPSL2714*, *BPSS0015*, *BPSS1265*, *BPSS1780*, *BPSS1818*, and *BPSS1860*, showed 15–71% wild-type replication at 24 h post infection (Supplementary Fig. 4d, Supplementary Table 1). Significant defects were observed at 24 h post infection for the *BPSL0097*, *BPSL2714*, *BPSS0015*, *BPSS1265*, *BPSS1780*, *BPSS1818*, and *BPSS1860* mutants (Supplementary Table 1). All 11 hypothetical genes have hundreds of orthologs in *Burkholderia* strains signifying that they may have conserved functions (Supplementary Table 1). In vitro growth and complementation analyses show that the defects during intracellular replication are not caused by reduced in vitro fitness or polar effect of these mutations, which validates these genes as virulence factors (Supplementary Fig. 5a-b).

To further evaluate the roles of these genes in *Bp* pathogenesis, we employed multiple established infection models including HEK293T plaque formation, live-cell imaging of RAW264.7 cell infection, and acute melioidosis infection in BALB/c mice^26,46^. Five of the mutants, *BPSL1126*, *BPSL1390*, *BPSL1422*, *BPSL2714*, and *BPSS1780*, showed disease progression comparable to wild-type *Bp*, indicating that even though they showed attenuation in the cell infection models (Supplementary Fig. 4c and 4d), these genes are not required for pathogenesis in vivo (Supplementary Fig. 5c). The *BPSS1265* mutant showed delayed morbidity with 60% survival of mice over the duration of the study, suggesting that this gene could be important for acute infection but attenuation does not extend to chronic forms of murine melioidosis (Supplementary Fig. 5c). The *BPSL0636* mutant showed significant attenuation in cell culture, marked by a significant reduction in plaque diameter during infection of HEK293T cells (Supplementary Fig. 6b). In RAW264.7 cells, we observed that the *BPSL0636* mutant replicates to wild-type *Bp* levels during the early stages of infection, but detected a major delay in the spread and/or fusion to host cells during late stages of infection (Supplementary Table 1, Supplementary Movie 2). In addition to attenuation in two drastically different cell models, mice infected with the *BPSL0636* mutant survived the entire length of the in vivo study, highlighting the importance of this gene in *Bp* pathogenesis (Supplementary Fig. 6c). Eighty percent of surviving mice completely cleared the *BPSL0636* mutant, while a single mouse had residual infection in the lungs (Supplementary Fig. 6c). This suggests that the *BPSL0636* mutant could serve as a base strain for potential live-attenuated vaccines against melioidosis. Beyond the defect in invasion and complete attenuation of the *BPSL0636* mutant in BALB/c mice, there was no obvious indication as to the pathogenic mechanism of this gene during infection (Supplementary Table 1, Supplementary Fig. 6c). As described below, several other mutants, *BPSL0097*, *BPSS1860*, *BPSS1818*, and *BPSS0015*, showed similar levels of attenuation in multiple cell lines and BALB/c mice, and additional experiments alluded to their possible pathogenic mechanisms during infection.

### Discovery of two attachment proteins

**Fig. 2:**
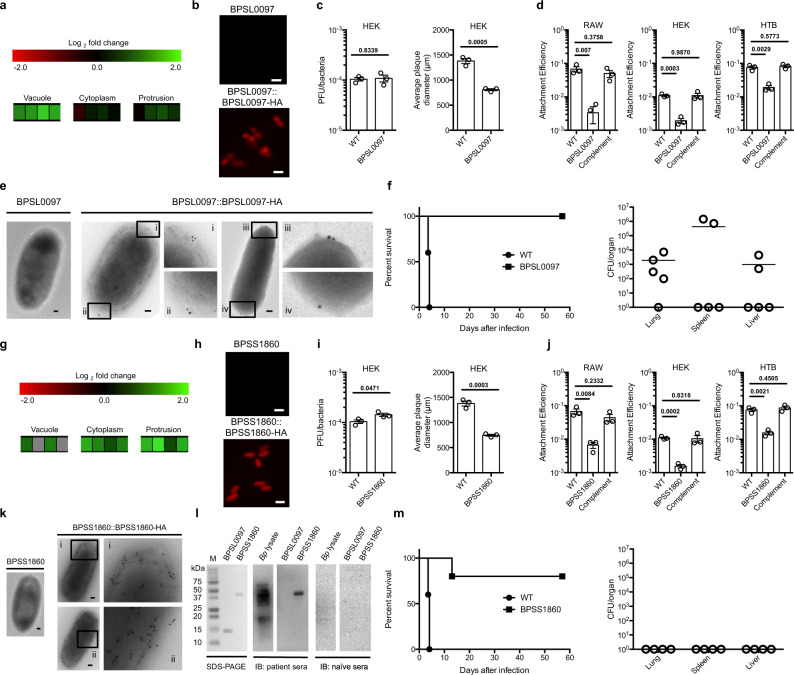
Characterizations of BPSL0097 and BPSS1860 mutants. **a** The *BPSL0097* gene is upregulated within the macrophage vacuole. First three boxes of each infection stage represent three biological replicates, the fourth represents the mean. **b** Immunofluorescence to a *BPSL0097*-*HA* fusion strain suggests that BPSL0097 is located on the surface of *Bp* while the *BPSL0097* mutant strain showed no signal. Scale bar = 1 μm. **c** Numbers of plaques formed (PFU) by the *BPSL0097* mutant in HEK293T monolayer (*n* = 3) is comparable to wild-type *Bp* indicating no defect in host cell invasion; however, reduced plaque diameters indicate overall infection defects. **d** The *BPSL0097* mutant showed reduced attachment efficiencies compared to wild-type *Bp*, suggesting a role in attachment to multiple host cell lines (*n* = 3). **e** TEM confirms the location of BPSL0097 to be the surface of *Bp* and further isolates its location to the poles of the cell. Scale bar = 100 nm. **f** The *BPSL0097* mutant is attenuated in BALB/c mice (*n* = 5) when infected via the intranasal route. Bacterial burdens of surviving mice suggest that the *BPSL0097* mutant persists in vivo. **g** *BPSS1860* gene is upregulated during cell infection relative to in vitro condition. First three boxes of each infection stage represent three biological replicates, the fourth represents the mean. **h** Immunofluorescence to a *BPSS1860*-*HA* fusion strain suggests that BPSS1860 is located on the surface of *Bp* while the *BPSS1860* mutant strain showed no signal. Scale bar = 1 μm. **i** Numbers of plaques formed (PFU) by the *BPSS1860* mutant in HEK293T monolayer (*n* = 3) is comparable to wild-type *Bp* but show reduced plaque diameters indicating an overall infection defect. **j** The *BPSS1860* mutant showed reduced attachment efficiencies compared to wild-type *Bp*, suggesting a role in attachment (*n* = 3). **k** TEM confirms that BPSS1860 is located on the surface of *Bp* and that it is distributed throughout the entire cell length. Scale bar = 100 nm. **l** Antibodies against purified BPSS1860 were detected via immunoblot (IB) in pooled melioidosis patients’ serum samples (*n* = 7). M, Precision Plus Protein Standard (Bio-Rad). **m** The *BPSS1860* mutant is attenuated in BALB/c mice (*n* = 5) when infected via the intranasal route. The *BPSS1860* mutant was completely cleared in all surviving mice. Data in bar graphs represent means ± s.e.m. and analyzed via two-sided unpaired *t*-test. *P* values presented above relevant comparisons.

Mutants of *BPSL0097* and *BPSS1860* showed an attenuated phenotype when tested in HEK293T cells (Fig. 2c and i, respectively, indicated by reduced plaque sizes) and the BALB/c model of melioidosis (Fig. 2f and m, respectively, marked by 100% and 80% survival). These genes are upregulated in the initial stage of cell infection and constitutively expressed throughout the *Bp* TRANSITome, respectively (Fig. 2a and g). As attachment to host cells occurs at the initial stages of infection before internalization, we investigated if these predicted outer membrane proteins have a role during this process. Analysis of the predicted coding regions of BPSL0097 and BPSS1860 indicate that each have signal sequences suggesting that they are secreted proteins and could be presented on the cell surface^47^. In conjunction with this observation, mutants of *BPSL0097* and *BPSS1860* exhibited a significant reduction in attachment efficiency in three cell lines, RAW264.7, HEK293T, and HTB11 cells, strongly indicating an attachment function associated with these two genes (Fig. 2d and j, respectively). A double mutant attaches at 4% of wild-type *Bp*, a further reduction from the 5 or 10% of single *BPSL0097* or *BPSS1860* mutants (*P* = 0.0512, *P* = 0.0101, respectively, comparing the double to single mutants) indicating that other *Bp* attachment factors are present. Although the loss of both *BPSL0097* and *BPSS1860* did not result in a complete abolishment of *Bp* attachment to host cells, the additional reduction to attachment efficiency in the double mutant may suggest that these adhesins operate separately. To validate that these proteins are involved in attachment, we compared wild-type *Bp* to each mutant via live-cell imaging. Wild-type *Bp* moves through the extracellular milieu and remains in contact for an extended period of time when encountering host cells (Supplementary Movie 3). In contrast, *BPSL0097* or *BPSS1860* mutants come into contact with host cells for a limited period of time, further validating these proteins as host cell attachment factors (Supplementary Movies 4 and 5, respectively). While our observations indicate that both proteins are essential for the initial stages of attachment, the role of BPSS1860 during the later stages has not been determined as it is expressed throughout the TRANSITome.

To confirm the prediction that BPSL0097 and BPSS1860 are presented on the outer surface of *Bp*, we generated complemented strains expressing hemagglutinin (HA) tagged fusion proteins (*BPSL0097*::*BPSL0097*-*HA* or *BPSS1860*::*BPSS1860*-*HA*). The HA-tagged fusion strains were stained positively, via immunofluorescence (IF) with an anti-HA antibody, confirming that these proteins are located on the surface of *Bp* (Fig. 2b and h). Because BPSL0097-HA appears to be located on the periphery of the bacterium and exhibited higher fluorescence signals on the poles (Fig. 2b), we proceeded with immunogold labeling (IG) and transmission electron microscopy (TEM) to gain a better resolution of each protein’s distribution on the bacterial surface. BPSL0097-HA showed localization limited to the poles of *Bp*, largely agreeing with the IF result (Fig. 2e). On the contrary, BPSS1860-HA showed an even distribution across the bacterial surface via IF (Fig. 2h) and this was validated by IG TEM (Fig. 2k). Since these two surface proteins are required for full pathogenesis in vivo (Fig. 2f and m), we further evaluated their immunogenicity in clinical melioidosis via immunoblot against serum from patients with melioidosis. While BPSL0097 showed no reaction to patient sera, purified BPSS1860 was recognized specifically by pooled patient sera demonstrating its potential as a diagnostic target (Fig. 2l). The lack of immunogenicity to BPSL0097 is likely due to low presentation on the bacterial cell surface, lack of immunogenic protein sequence, or a representation of the number of sera samples pooled (*n* = 7). Taken together, the data presented here highlights the discovery of these attachment proteins, BPSL0097 and BPSS1860, as virulence factors independently required for the progression of *Bp* infection.

### BPSS1818 modulates host cell tubulin

**Fig. 3:**
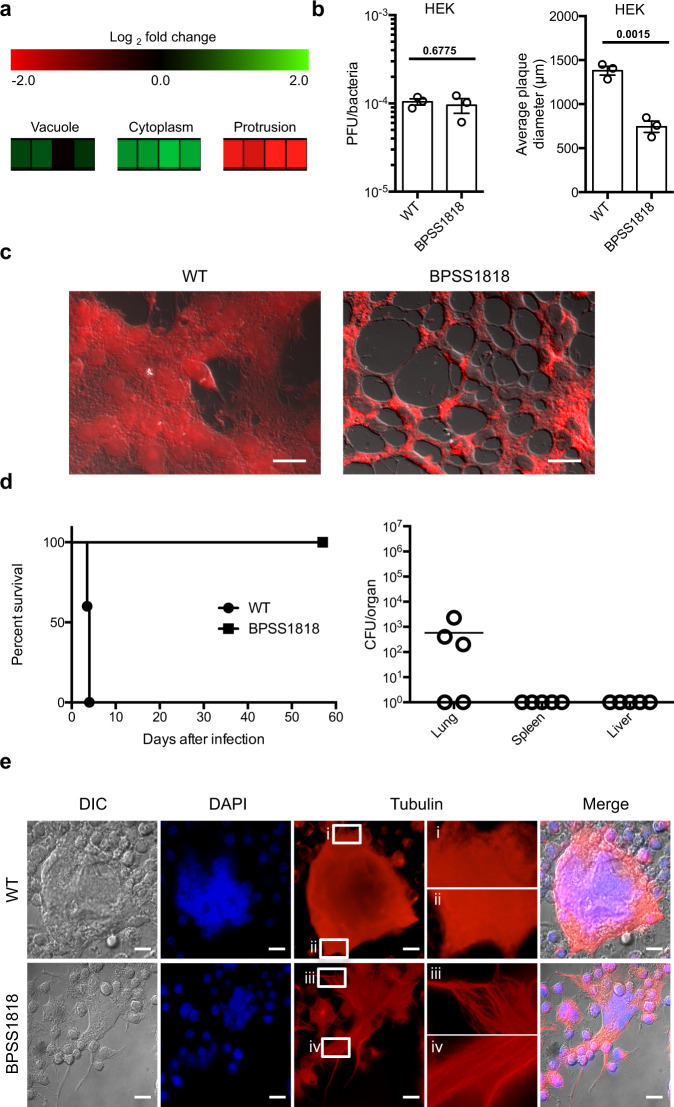
Characterizations of *Bp* mutant BPSS1818. **a** *BPSS1818* gene is upregulated within the macrophage cytosol, then significantly downregulated during the protrusion stage of infection. First three boxes of each infection stage represent three biological replicates, the fourth represents the mean. **b** Number of plaques formed (PFU) by the *BPSS1818* mutant is comparable to wild-type *Bp*, indicating no defect in host cell invasion; however, reduced plaque diameters were observed indicating defect in infection spread (*n* = 3). Data represent means ± s.e.m. and analyzed via two-sided unpaired *t*-test. *P* values presented above relevant comparisons. **c** Fused RAW264.7 cells infected with the *BPSS1818* mutant show extended host cell cytoskeleton and distended MNGCs compared to wild-type *Bp*. Scale bars = 5 μm. **d** The *BPSS1818* mutant is completely attenuated in BALB/c mice (*n* = 5) when compared to wild-type *Bp*. Bacterial burdens were only observed at low levels in lungs of three mice. **e** RAW264.7 cells infected with the *BPSS1818* mutant showed elongated microtubules with pronounced fibers when magnified in contrast to wild-type *Bp* infected cells. Scale bars = 10 μm.

Highly expressed in the cytoplasm (Fig. 3a), a mutant of *BPSS1818* also showed changes in phenotype during in vitro and in vivo infections (Fig. 3). In vitro growth and complementation analyses in Fig. S5b show that these defects are not caused by reduced in vitro fitness, secondary mutation, or polar effect, indicating that mutation in *BPSS1818* is responsible for the observed phenotypes in Fig. 3. The mutant of *BPSS1818* was highly attenuated in RAW264.7 cells (Supplementary Fig. 4d and Supplementary Table 1) as well as in HEK293T cells (Fig. 3b). More importantly, the *BPSS1818* mutant was 100% attenuated in BALB/c mice, indicating that it is essential for in vivo pathogenesis (Fig. 3d). To better investigate the associated pathogenic function of this gene, we revisited our in vitro infection models. During infection of RAW264.7 cells with the *BPSS1818* mutant, we noticed a major phenotypic change in the overall monolayer morphology when compared to cells infected with wild-type *Bp* (Supplementary Movie 6, Fig. 3c). Cells infected with the *BPSS1818* mutant appear to be varied in overall cytoskeletal structure after extensive host cell fusion, and the ‘stretched-out’ MNGCs were unable to collapse into a spherical structure (Supplementary Movie 6, Fig. 3c). This finding suggests that BPSS1818 modulates the host cell cytoskeleton leading to this phenotype in the absence of *BPSS1818*. Modulation of the host cell cytoskeleton components myosin, actin, and tubulin by BPSS1818 was determined by IF. There were no morphological changes in myosin and actin filaments, between RAW264.7 macrophage cells infected with the *BPSS1818* mutants and wild-type *Bp*. On the other hand, RAW264.7 cells infected with the *BPSS1818* mutant showed variations in the morphology of tubulin, as noted by elongated polymers when compared to the wild-type *Bp* infected host cells (Fig. 3e). BPSS1818 is a predicted inner membrane protein suggesting that the modulation of tubulin is indirect and possibly requires other components. Overall, the data support the contention that BPSS1818, which indirectly modulates host cell tubulin, is required for full pathogenesis of *Bp*. *Bp* has been shown previously to modulate host cell actin^30^, but, to our knowledge, this is the first report showing that *Bp* affects tubulin during infection of the host cell.

### *Bp* evades autophagy clearance via BPSS0015

**Fig. 4:**
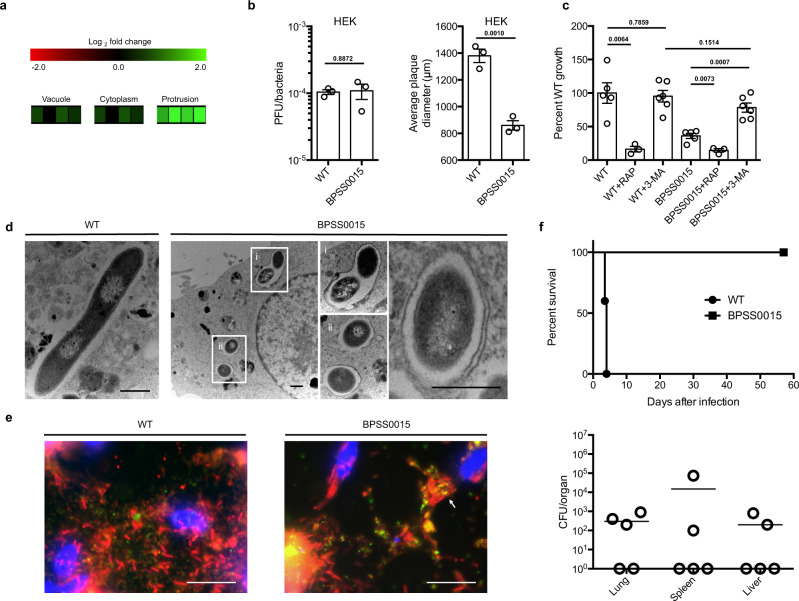
Characterization of *Bp* mutant BPSS0015. **a** *BPSS0015* is upregulated at the protrusion stage during infection. First three boxes of each infection stage represent three biological replicates, the fourth represents the mean. **b** Number of plaques formed (PFU) by the *BPSS0015* mutant is similar to wild-type *Bp*, indicating no defects in host cell invasion; however, reduced plaque diameters were observed, suggesting defects during the progression of infection (*n* = 3). **c** Rapamycin-induced (RAP) autophagy reduced the survival of both wild-type *Bp* and the *BPSS0015* mutant (*n* = 3), whereas the inhibition of autophagy with 3-methyladenine (3-MA) significantly restored intracellular replication of the BPSS0015 mutant to wild-type level (*n* = 6). **d** Representative TEM images of macrophages infected with the *BPSS0015* mutant show each bacterium surrounded by membrane structures. Scale bars = 1 μm. **e** Infected HEK293T cells stably expressing GFP-LC3 show co-localization of the autophagy marker, LC3 (green), with the *BPSS0015* mutant (red), indicated by the white arrow, and no co-localization with wild-type *Bp* (red). Scale bars = 10 μm. **f** The *BPSS0015* mutant is completely attenuated in BALB/c mice (*n* = 5) but bacterial burdens of surviving mice suggest that this mutant persists in vivo. Data in bar graphs represent means ± s.e.m. and analyzed via two-sided unpaired *t-*test. *P* values presented above relevant comparisons.

The *Bp* TRANSITome revealed that the *BPSS0015* gene was highly expressed in the protrusion stage during host cell infection, suggesting its importance for later steps of the infection lifecycle (Fig. 4a). In vitro growth and complementation analyses in Fig. S5b show that these defects are not caused by reduced in vitro fitness, secondary mutation, or polar effect, suggesting that mutation in *BPSS0015* is responsible for the observed phenotypes in Fig. 4. A mutant of this gene showed a significant decrease in intracellular replication and plaque sizes in RAW264.7 and HEK293T cells, respectively (Supplementary Fig. 4d, Fig. 4b). When used to infect BALB/c mice at a lethal dose, all mice survived during the entire study period, indicating that the *BPSS0015* gene is an essential virulence determinant for *Bp* pathogenesis in vivo (Fig. 4f). Unlike mutants of *BPSS1818*, *BPSL0636*, and *BPSS1860*, the *BPSS0015* mutant was able to persist and disseminate to the spleen and liver of surviving mice (Fig. 4f). Upon closer examination using the cell infection model, the *BPSS0015* mutant appeared to be trapped in membrane-bound structures (Fig. 4d). To confirm this observation, RAW264.7 cells infected with wild-type *Bp* and the *BPSS0015* mutant were processed for TEM. The *BPSS0015* mutant was encompassed by single and double membrane-bound vacuoles, properties of autophagy clearance, while wild-type *Bp* are not associated with any membrane-bound structures within the cytoplasm (Fig. 4d). A common marker of autophagy, LC3^48^, was then chosen to evaluate if BPSS0015 was linked to autophagy evasion. HEK293T cells stably expressing LC3-GFP were infected with the *BPSS0015* mutant and wild-type *Bp* to assess variations in co-localization with the host cell LC3. While wild-type *Bp* does not associate with the LC3-GFP puncta, the *BPSS0015* mutant shows co-localization (Fig. 4e). Although a previous report showed that wild-type *Bp* does associate with LC3 in RAW264.7 macrophages at very low levels during the first 6 h of infection^49^, we did not see that association in HEK293T cells at 24 h post infection. In addition, type three secretion system effectors, BopA and BipD, were previously identified in the reduction of LC3-associated phagocytosis during early stages of infection^36^, indicating that *Bp* possesses multiple mechanisms to avoid intracellular clearance during various stages of host cell transit. These data taken together imply that the *BPSS0015* mutant is unable to avoid host cell autophagy clearance during the late stages of infection. To further test this hypothesis, we utilized two chemical modulators that control the level of host cell autophagy, rapamycin, and 3-methyladenine^49^. Rapamycin, a stimulator of host cell autophagy, reduced the intracellular burden of wild-type *Bp* and the *BPSS0015* mutant compared to the control infection (Fig. 4c). An infection supplemented with 3-methyladenine, a suppressor of host cell autophagy, showed no effect on wild-type *Bp* intracellular replication, further supporting the ability of wild-type *Bp* to avoid host cell autophagy clearance (Fig. 4c). On the contrary, the *BPSS0015* mutant aided by 3-methyladenine was able to recover its ability to replicate within the host cells (Fig. 4c). Taken together, the data indicate that *Bp* is able to avoid host cell autophagy during the late stages of infection and that the *BPSS0015* gene is involved in this autophagy evasion mechanism. Although previous studies have shown that during initial stages of infection (<6 h post infection) *Bp* avoids LC3-associated phagocytosis^36,49^, this is the first investigation to identify a gene involved in *Bp* evasion of autophagy during late stages of infection.

### Model of *Bp* intracellular pathogenesis

The current understanding of intracellular *Bp* infection has been based on numerous studies identifying virulence mechanisms for attachment, invasion, replication, and spread within host cells^23–32^. While other transcriptomic analysis of *Bp* has focused on various in vitro growth conditions^41^ or populations within a host^38,50,51^, our study is the first to focus on the microenvironments that a single *Bp* cell encounters during intracellular infection. The present work has added an extensive amount of information to the *Bp* intracellular lifecycle, but it is not without its limitations. We only investigated three time points during *Bp* intracellular transit even though gene expression flux is likely to occur continuously and dynamically. Investigating additional time points during intracellular transit could generate a higher resolution picture of *Bp* pathophysiology within the host. Although validated with RT-PCR, we recognize that some variations in gene expression could be due to amplification bias, as inconsistent amplification of some genes is hard to avoid for single-cell transcriptomes. The current study focused on hypothetical proteins that are consistently expressed in at least one spatial region of the host. While this approach yielded results, it also neglected to account for genes that show variable expression within each spatial region, a category of gene that could lead to valuable insights in the individuality of single prokaryotic cells within the context of infection. Last, this work focused on a *Bp* intracellular infection model within RAW264.7 murine macrophages and could be extended into in vivo infections of mice and clinical samples.

**Fig. 5:**
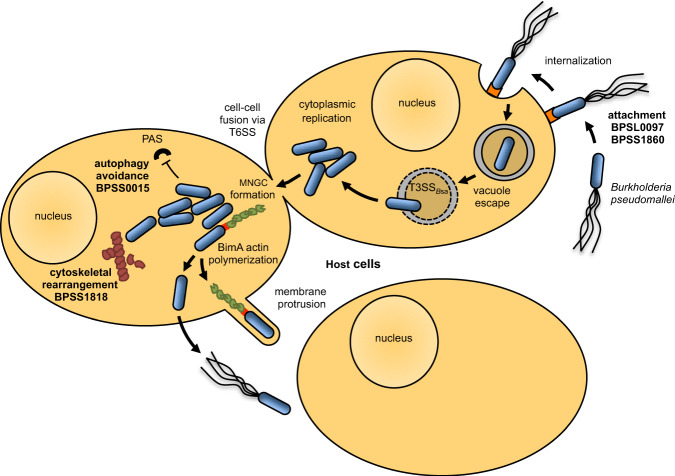
Model of *Bp* intracellular pathogenesis. *Bp* attaches unknown receptors (orange) on host cells, using surface attachment proteins BPSL0097 and BPSS1860. After internalization, *Bp* escapes the vacuole utilizing the *Burkholderia* secretion apparatus (T3SS_Bsa_)^23^ and moves within the host cell using its secondary flagella locus^26^ or by polymerizing host cell actin (green) with bacterial BimA (red boxes on the bacterial cell pole)^30^. BPSS1818 facilitates changes in host cell cytoskeletal structure through modulation of tubulin (maroon), while BPSS0015 contributes to autophagy evasion. PAS: pre-autophagosomal structure. Bacteria replicate intracellularly and spread to neighboring cells by actin-based membrane protrusion and by promoting cell fusion to form multinucleated giant cells (MNGC) using a Type VI Secretion System (T6SS)^24,27,28^.

In summary, we have dissected and defined the intracellular TRANSITome of *Bp*, a globally significant pathogen. Thousands of known and unknown genes and pathways undergo dynamic gene-expression flux as *Bp* transits through distinct environmental niches in host cells. The *Bp* TRANSITome led to the discovery of several virulence factors that are required for complete *Bp* pathogenesis. Comprehensive screens of 191 mutants and functional characterizations determined potential functions for some of these virulence factors during *Bp* infection of host cells. Based on the data presented, we suggest assigning functions to four of these virulence factors: BPSL0097 and BPSS1860 as surface attachment proteins, BPSS1818 as a modulator of host cell tubulin, and BPSS0015 as a factor involved in evasion of host cell autophagy (Fig. 5). These virulence determinants have the potential to be exploited as therapeutic targets and vaccines against melioidosis. Beyond the findings presented in this research, the TRANSITome data (Supplementary Dataset 1) could be mined for information about other intracellular *Bp* processes. Finally, this approach of single prokaryotic cell transcriptomics can be applied toward broad scientific discovery through expanding the possibilities for a better understanding of polymicrobial diseases, symbiosis, transcriptomics of unculturable microbes, host-pathogen interactions, and dynamics within microbiomes.

## Methods

### Bacterial strains and eukaryotic cell lines, media, and culturing conditions

All manipulation of *Bp* was conducted in a CDC-approved and -registered BSL3 facility at the University of Hawaiʻi at Mānoa with all experiments approved by the Institutional Biosafety Committee (reference number: 16-07-004-585-1R) and were performed using BSL3 practices following recommendations set forth in the BMBL, 5th edition. *Escherichia coli* EPMax10B-*lacI*^*q*^-*pir*^52^ was routinely used as a cloning strain. The *Bp* wild-type strains, K96243 and 1026b, and their derivatives were cultured in LB or 1x M9 minimal media supplemented with 20 mM glucose (MG). For selection of glyphosate resistance gene (*gat*) in *E. coli* and *Bp*, MG medium supplemented with 0.3% (w/v) glyphosate was used. Murine macrophage cell line (RAW264.7, ATCC TIB-71), human embryonic kidney cell line (HEK293T, ATCC CRL-3216), and human neuroblastoma cell line (SK-N-SH, ATCC HTB-11) were used in this study for *Bp* infection. All cell lines are from in-house collections of widely used and commercially available cell lines, originally obtained from ATCC. The eukaryotic cell cultures were grown in DMEM medium (Hyclone) supplemented with 10% FBS (Hyclone) at 37 °C with 5% CO_2_, and the Antibiotic-Antimycotic reagent (Invitrogen) was added at 1× concentration for cell culture maintenance but omitted during *Bp* infection studies.

### Molecular reagents and methods

All molecular reagents and methods were used as previously described^1,35^. All oligonucleotides used in this manuscript are listed in Supplementary Dataset 2.

### Macrophage infection with fluorescence-tagged *B. pseudomallei*

The *Bp* wild-type strain K96243 was labeled with red- and yellow-fluorescence proteins for easy visualization during macrophage infection. Briefly, the pUC57-PS12-*yfp*^53^ was digested with *Nco*I, blunt-ended, and the *yfp* gene was ligated with the mini-Tn7-*gat*-*rfp*^53^ backbone digested with BamHI and blunt-ended. The *yfp* gene is in the same orientation as the *rfp* gene in the resulting plasmid and both genes are driven by a constitutive promoter in *Bp*, PC_*S12*_^54^. The mini-Tn7-*gat-rfp-yfp* plasmid was then conjugated into K96243 along with the helper plasmid pTNS3-*asd*_*Ec*_^55^. *Bp* with insertion of the mini-Tn7 plasmid at the *attTn7* site was confirmed by PCR as previously described^52^. Stable expression of the RFP and YFP proteins was confirmed and used for macrophage infection study.

The macrophage infection with *Bp* was carried as follows. First, the RAW264.7 cells were seeded onto 0.17 mm PET membrane-coated MembraneSlides (Carl Zeiss) that were pretreated with UV and then 150 μg/ml poly-L-lysine. The RFP-YFP-tagged *Bp* strain was grown to mid-log phase (OD_600_ ~ 0.8) and diluted to ~4 × 10^5^ CFU/ml in DMEM medium with 10% FBS. RAW264.7 cells were infected at a multiplicity of infection (MOI) of 0.2 for 30 min and extracellular *Bp* were washed away with 1× PBS. Fresh DMEM medium supplemented with 10% FBS was then added to the membrane slides without antibiotics to ensure a valid comparison to the control. At 1, 2, 6 h post infection, correlating to the vacuole, cytoplasm, and protrusion stages, respectively, the membrane slides were washed with 1× PBS and immediately fixed with 1% (w/v) paraformaldehyde for 5 min followed by 70% (v/v) ethanol for 30 min. To obtain the control *Bp* cells as the baseline for microarray comparison, the same diluted *Bp* culture as above was incubated in the DMEM medium with 10% FBS for 1 h, harvested by centrifugation, and resuspended in 1% (w/v) paraformaldehyde for 5 min. The fixed bacteria were then smeared onto the membrane slides, treated with 70% (v/v) ethanol for 30 min.

To visualize *Bp* at different stages during macrophage infection, we stained the macrophage plasma and vacuolar membranes and actin with FM 4-64FX lipophilic dye and Oregon Green 488 Phalloidin (Thermo Fisher Scientific), respectively. The fluorescent images were obtained on a Zeiss PALM laser catapulting system with ×100 oil immersion objective. Single *Bp* cells from the various stages (vacuole, cytoplasmic replication, and protrusion) of infection were cut by the focused laser and catapulted with unfocused low-intensity laser into the 0.2 ml PCR tube lid containing lysis buffer^1,35^. Cells within the cytoplasm and protrusion stages carried actin tails but were differentiated by the time post infection and visual location in respective intracellular niche. For the control condition (*Bp* grown in DMEM), a total of nine *Bp* cells were catapulted into the same PCR tube before processing. Single *Bp* cells and pooled control *Bp* cells were processed using previously described methods^1,35^.

### Two-color microarray and data analysis

Transcriptomic analysis was carried out with the *B. mallei*/*pseudomallei* 70mer oligo arrays kindly provided by the J. Craig Venter Institute. Each *Bp* cell was estimated to contain <2 pg of total RNA^1,35^. Single *Bp* cells isolated directly from host (Fig. 1a–d) were lysed, and the cDNA synthesis and amplification from single bacterium total RNA were performed^1,35^. The amplified cDNA was labeled with Cy3 or Cy5 dye and hybridized to the *B. mallei*/*pseudomallei* 70mer oligo arrays following the established protocols as described^1,35,56^. Microarray slides were scanned in a GenePix 4000 microarray scanner with GenePix Pro software 5.1. Individual TIFF images from each channel were processed with Spotfinder software 3.2.1 to generate the raw data. The raw data were then normalized using MIDAS software 2.21 with low-intensity filtering, LOWESS normalization, standard deviation regularization, and in-slide replicate analysis. Finally, the normalized data in technical replicates were combined, using MEV software 4.5.1, to generate gene fold-change data (*P* ≤ 0.05) when comparing one single bacterium during macrophage infection to the in vitro control. The biological replicates for each infection stage were also merged using MEV software to identify the genes that were consistently up- or downregulated through one-way ANOVA with multiple comparisons (*P* ≤ 0.05). All heat maps are presented in a green-black-red color gradient; green color indicates upregulation and red color indicates downregulation, when the gene expression in each infection stage was compared to the control condition. Gray boxes represent the lack of expression data in a technical replicate for that gene. Reference genome of K96243 type strain was used and a total of 5797 genes were analyzed^33^.

### Gene assignment and pathway designation

Gene description, function prediction, and functional category assignment were assisted for some genes using *Burkholderia* Genome database (http://www.burkholderia.com)^33^ and Kyoto Encyclopedia of Genes and Genomes (KEGG)^57^.

### Real-time RT-PCR

Validation of microarray data with real-time RT-PCR was performed using Taqman probe. Three housekeeping genes, *BPSL0602*, *BPSL2502*, and *BPSS2061*, that have consistent expression levels across all conditions tested were chosen based on our microarray data, as well as published transcriptomic data^41^. *BPSS1511*, *BPSL1528*, and *BPSL1064* showed differential expression in the TRANSITome data with upregulation in the vacuole, cytoplasm, and protrusion, respectively, and similar expression results were observed using RT-PCR. Oligos for real-time RT-PCRs were designed using Integrated DNA Technologies Primer Quest software (http://www.idtdna.com/) and the sequences were included in Supplementary Table 2. Each real-time PCR reaction contains 120 nM of each forward and reverse oligos, and 12 nM of probe. Real-time PCR was performed in the iCycler iQ (Bio-Rad) with the following steps: denaturation (95 °C for 10 min), 55 cycles of amplification, and quantification (95 °C for 20 s and 65 °C for 45 s). Supermixes for all reactions were made and aliquoted into sub-supermixes for each gene assayed. Eight RT-PCR reactions were done for each gene and condition, performed for three single *Bp* cells as biological replicates. Data were processed and fold-changes were calculated following the method of Peirson et al.^58^.

### Engineering of the *Bp* 1026b virulence factor mutants

Chromosomal mutant knock-outs were attempted for 206 spatially upregulated *Bp* hypothetical genes in wild-type 1026b strain, using λ-RED recombineering as previously described^39^. Mutations were generated by insertion of glyphosate resistance gene *gat* for selection, along with *pheS* gene for counter-selection for subsequent complementation^39^. We successfully mutated 191 of the 206 genes attempted. All mutants were verified using PCR.

### Engineering of the *Bp* 1026b virulence factor complements

The 11 mutants that were determined to be defective in the intracellular replication assay (Supplementary Table 1) were complemented and tested via intracellular replication assay in Fig. S5b. The complementation was done by reintroducing a single copy of the gene at their native loci in the corresponding mutant, using λ-RED recombineering as previously described and counter-selecting for the loss of *pheS* gene in the resulting complemented strain^39^. All complements were verified using PCR.

### Growth curves of *B. pseudomallei* 1026b mutants

Growth curves were initiated by growing the 1026b wild-type and various mutant strains overnight, then diluting 200× into fresh LB. The 96-well plate was incubated at 37 °C with shaking in the BioTek ELx808IU and measuring the OD_600_ every 30 min for 30 h. Each growth curve was done in triplicate and average was presented with the standard error of the mean (s.e.m.).

### Purification/immunoblot of BPSL0097 and BPSS1860

BPSL0097 and BPSS1860 were purified using nickel affinity chromatography. BPSL0097 was purified with an N-terminal His_6_-tag from pViet^59^ in *E. coli* ER2566 codon plus strain under native condition, and BPSS1860 was purified with an N-terminal His_6_-tag from pViet^59^ in *E. coli* ER2566 codon plus strain under denatured condition with 8 M urea. Purified BPSL0097 and BPSS1860 were run on a SDS-PAGE gel and blotted to a PVDF membrane. Immunoblots were carried out using the Western Breeze blocker/diluent and protocol (Thermo Fisher). A pool of melioidosis patient sera (*n* = 7) and naive sera (*n* = 2) was de-identified and used to probe the blotted PVDF membrane (1:40 dilution). A secondary goat anti-human Ig-HRP antibody (1:10,000 dilution, Invitrogen, Catalog No. AHI0704, Lot No. 1228096) and Novex™ ECL Chemiluminescent Substrate Reagent Kit (Thermo Fisher) was used for detection.

### Cell infection assays

Intracellular replication assays were carried out using a modified aminoglycoside protection assay. RAW264.7 cells were seeded at ~80% confluence, infected at an MOI of 1 for 1 h, washed with 1× PBS, and then fresh DMEM + FBS containing 750 μg/ml amikacin and 750 µg/ml kanamycin was added. At various time points post infection, monolayers were washed two times with 1× PBS, lysed with 0.2% Triton X-100 in PBS, and dilutions of lysates were plated onto LB to enumerate intracellular bacteria. Attachment assays were carried out with various cell lines at an MOI of 1. For attachment efficiency test, infection was initiated similarly as the intracellular replication assay, and at 1 h post infection (hpi) the bacteria-containing medium was removed, the monolayers were washed three times with PBS, and lysed with 0.2% Triton X-100 in PBS, diluted, plated onto LB to enumerate attached bacteria. The attachment efficiency was determined by dividing the attached number by the initial number of infecting bacteria. All experiments were carried out in triplicate and error bars represent the s.e.m.

Plaque assays were carried out in confluent monolayers of HEK293T cells in 24-well plates. *Bp* 1026b strains were used to infect monolayers at an MOI of 1. After 1 h, monolayers were washed with 1× PBS and overlaid with DMEM + FBS supplemented with 1.2% low-melt SeaPlaque agarose (Lonza) and 750 μg/ml amikacin and 750 µg/ml kanamycin. At 24 hpi, monolayers were fixed with 4% paraformaldehyde (PFA) in 1× PBS for 45 min. Monolayers were stained with a 1% crystal violet solution for ease of viewing. Plaques were viewed with a Zeiss AxioObserver D1 and the accompanying AxioVision 64 bit 4.9.1 software was used to measure plaque diameter. Plaque assays were carried out in triplicate and 10–20 plaques per replicate were measured for comparison.

Chemical modulation of autophagy was carried out as previously described with minor modifications^49^. Briefly, RAW264.7 cells were treated with either 4 µM rapamycin or 10 mM 3-methyladenine 1 hpi and bacterial cell counts determined at 24 hpi as described above.

### Immunofluorescence and transmission electron microscopy

The BPSL0097 and BPSS1860 mutant strains were complemented with translational HA (human influenza hemagglutinin)-tagged *BPSL0097*, or *BPSS1860*, respectively. Mutants and the complemented strains were stained with a primary antibody of mouse anti-HA conjugated to Alexa Fluor 594 (1:200 dilution, Thermo Fisher Scientific, Catalog No. A-21288, Lot No. 1740027), followed by secondary goat-anti-mouse antibody Alexa Fluor 488-10 nm colloidal gold conjugate (1:50 dilution, Thermo Fisher Scientific, Catalog No. A-31561, Lot No. 1348715). Labeled bacterial cells were visualized using a Zeiss D1 observer fluorescence microscope and a 120 kV Hitachi HT7700 digital transmission electron microscope.

For visualization of the *BPSS0015* mutant infected macrophage cells, 60-mm tissue culture dishes were treated with 150 μg/ml poly-L-lysine and RAW264.7 murine macrophages were seeded and allowed to attach overnight. Wild-type *Bp* 1026b and the *BPSS0015* mutant strain were used to infect the monolayers as described above. At 24 hpi, the media was removed and the monolayers were fixed for 2 h with 2.5% glutaraldehyde in 0.1 M sodium cacodylate buffer at pH 7.4. The dishes were washed twice with 0.1 M cacodylate buffer for 20 min each. Samples were post-fixed in 1% osmium tetroxide in 0.1 M cacodylate buffer for 1 h then dehydrated in a graded ethanol series. Epoxy resin was used to infiltrate samples and allowed to polymerize at 60 °C for 2 days. Samples were visualized using a 120 kV Hitachi HT7700 digital transmission electron microscope. Images were captured using an AMT XR-41 2048 × 2048 pixel bottom-mount high-resolution camera.

For visualization of LC3 co-localization to the *BPSS0015* mutant or wild-type *Bp*, HEK293T cells were transfected with pEGFP-LC3 (Addgene #21073) using Lipofectamine 2000 Reagent (Invitrogen) following the manufacturers protocol. Wild-type *Bp* 1026b and the *BPSS0015* mutant strain were used to infect, as described above, the monolayers stably expressing LC3-GFP. At 24 hpi, monolayers were fixed with 1% PFA for 1 h, permeabilized with 0.2% Triton X-100, stained with DAPI and FM 4-64FX lipophilic dyes, and visualized using Zeiss D1 observer fluorescence microscope.

For visualization of *BPSS1818* mutant infected macrophage cells, the infected monolayer was stained by red plasma membrane stain (Fig. 3c), or DAPI and β-tubulin antibody conjugated with Alexa Fluor 594 (1:50 dilution, Cell Signaling Technology, Catalog No. 7634S, Lot No. 1) (Fig. 3e). Images were captured with an AxioObserer D1 and accompanying Axiovision 4.9.1 software. Multi-color fluorescent images were captured with the multichannel fluorescence acquisition module of the Axiovision software. Images were deconvolved using the imageJ plugin Iterative Deconvolve 3D.

### Live-cell time-lapse imaging

Light microscopy of infected cell monolayers was carried out as described^53^, except for a few modifications. Glass bottom 12-well plates were obtained from MatTek Corporation and treated with 150 μg/ml poly-L-lysine. Monolayers were seeded and infected with bacteria in 200 μl of DMEM + FBS at an MOI of 10. After 1 h the medium containing bacteria was removed, the monolayers were washed two times with 1XPBS, and DMEM containing 750 μg/ml amikacin and 750 μg/ml kanamycin was added for the remainder of the experiment. Live-cell imaging was taken on an Olympus microscope equipped with the Weather Station incubation system at 37 °C with 5% CO_2_. Images were captured at 1 frame/5 min for 24 h. Images were compiled into videos using ImageJ.

### Animal studies

BALB/c mice between 4 and 6 weeks of age were purchased from Charles River Laboratory. Animals were anesthetized with 100 mg/kg ketamine plus 10 mg/kg xylazine and infected with *Bp* via the intranasal (i.n.) route^46^. Groups of mice (*n* = 5) were challenged with a dose of 4500 CFU (5× LD_50_ for wild-type *Bp* 1026b), monitored daily for disease symptoms, and euthanized according to pre-determined humane endpoints. The lungs, liver, and spleen of surviving mice were removed, homogenized, and serial diluted to determine bacterial burdens. Statistical differences in survival times were determined by Kaplan–Meier curves followed by the log-rank test.

### Ethics

The animal studies described in this manuscript were conducted in compliance with the NIH (National Institutes of Health) Guide for the Care and Use of Laboratory Animals and approved by the Institutional Animal Care and Use Committee at the University of Hawaiʻi at Mānoa (Protocol No. 10-1073). Melioidosis patient sera samples used in immunoblot analysis were de-identified. Blood samples were collected following informed consent under ethics approval provided by Townsville Hospital and Health Services, Australia (HREC/12/QTHS/213).

### Statistics and reproducibility

Microarray data were analyzed in MEV software 4.5.1. All other statistical analyses were completed in Prism software 6.0. All experiments were done in triplicate unless otherwise noted, and all the data points are shown in graphs for quantitative analyses and raw data provided in the source data file (Source_Data_File.xlsx). All qualitative microscopy experiments were done in triplicate and representative images were shown. All experiments were repeated independently at least once, and replications were successful.

### Reporting summary

Further information on research design is available in the Nature Research Reporting Summary linked to this article.

## Supplementary information

Supplementary Information

Description of Additional Supplementary Files

Supplementary Data 1

Supplementary Data 2

Supplementary Movie 1

Supplementary Movie 2

Supplementary Movie 3

Supplementary Movie 4

Supplementary Movie 5

Supplementary Movie 6

Reporting Summary

## Data Availability

The microarray datasets generated in this study have been deposited in NCBI’s Gene Expression Omnibus^60^ and are accessible through GEO Series accession number “GSE156938”. Gene description, function prediction, and functional category assignment was assisted for some genes using *Burkholderia* Genome database (http://www.burkholderia.com). All other relevant data supporting the key findings of this study are available within the article and its Supplementary Information files or from the corresponding author upon reasonable request. A reporting summary for this Article is available as a Supplementary Information file. Source data are provided with this paper.

## Source data

Source Data
