# Supplementary material for: The Burkholderia pseudomallei intracellular ‘TRANSITome’
Source: Nat Commun. 2021 Mar 26;12:1907. doi: 10.1038/s41467-021-22169-1 (PMC7998038; doi:10.1038/s41467-021-22169-1)
Supplement: Supplementary file 1 — Supplementary Information [file 41467_2021_22169_MOESM1_ESM.pdf]

Supplementary Information

The *Burkholderia pseudomallei* intracellular 'TRANSITome'

Yun Heacock-Kang[1], Ian A McMillan[1§], Michael H Norris[1,2,§], Zhenxin Sun[1], Jan Zarzycki-Siek[1], Andrew P Bluhm[1,2], Darlene Cabanas[1], Robert E Norton[3,4], Natkunam Ketheesan[5], Jeff F Miller[6], Herbert P Schweizer[7] & Tung T Hoang[1]

[1]School of Life Sciences, University of Hawai'i at Mānoa, Honolulu, Hawai'i, USA; [2]Present address: Department of Geography and Emerging Pathogens Institute, University of Florida, Gainesville, Florida, USA; [3]Townsville Hospital, Townsville, Queensland, Australia; [4]Faculty of Medicine, University of Queensland, Australia; [5]Science and Technology, University of New England, New South Wales, Australia; [6]Department of Microbiology, Immunology, and Molecular Genetics, and the California NanoSystems Institute, University of California, Los Angeles, California, USA; [7] Pathogen and Microbiome Institute, Northern Arizona University, Flagstaff, Arizona, USA; [§]contributed equally to this work

*Correspondence should be addressed to T.T.H. (tongh@hawaii.edu)

Supplementary Table 1| Attachment frequencies, invasion frequencies, and intracellular replication in RAW264.7 cells are reported with statistical significance for the 11 attenuated mutants. The annotations/functional predictions, predicted cellular locations, and presence in other *Burkholderia* species of these 11 mutants are presented.

Gene ID	Attachment frequency[1] (P value)[2]	Invasion frequency[1] (P value)[2]	Mutant intracellular replication[1] (P value)[2]				Annotation/functional prediction	Cellular location PSORTb 3.0	Orthologs[3]
			2hpi	6hpi	16hpi	24hpi			
BPSL0097	**5±3 (0.0070)**	55±10 (0.1145)	**17±12 (0.0196)**	50±25 (0.2302)	97±25 (0.9264)	**16±1 (<0.0001)**	Hypothetical protein	Unknown	627
BPSL0636	60±18 (0.2046)	**25±10 (0.0285)**	94±8 (0.7891)	175±66 (0.3486)	101±12 (0.9171)	71±28 (0.3739)	Hypothetical protein	Unknown	652
BPSL1126	**41±8 (0.0432)**	90±15 (0.7096)	**37±5 (0.0290)**	**0±0 (0.0161)**	93±9 (0.6915)	108±6 (0.3340)	Hypothetical protein	Cytoplasmic membrane	646
BPSL1390	**29±5 (0.0204)**	285±100 (0.1435)	71±34 (0.4970)	75±43 (0.6433)	107±1 (0.5136)	100±5 (0.9027)	Hypothetical protein	Unknown	278
BPSL1422	**33±6 (0.0260)**	40±10 (0.0550)	**25±2 (0.0153)**	25±25 (0.1012)	69±14 (0.1586)	95±7 (0.6401)	Hypothetical protein	Cytoplasm	652
BPSL2714	**44±6 (0.0431)**	70±5 (0.2193)	94±25 (0.8615)	25±25 (0.1012)	75±13 (0.2182)	**18±0 (<0.0001)**	Hypothetical protein	Cytoplasmic membrane	654
BPSS0015	52±22 (0.1740)	45±9 (0.0651)	44±15 (0.0783)	625±195 (0.0560)	138±10 (0.0628)	**15±1 (<0.0001)**	Hypothetical protein	Cytoplasmic membrane	642
BPSS1265	122±3 (0.3113)	150±9 (0.0835)	144±27 (0.2434)	200±66 (0.2302)	115±6 (0.3048)	**21±2 (0.0001)**	Hypothetical protein	Extracellular/unknown	651
BPSS1780	**35±10 (0.0372)**	100±36 (1.000)	**196±25 (0.0366)**	**275±50 (0.0352)**	132±34 (0.4235)	**17±1 (<0.0001)**	Hypothetical protein	Cytoplasmic membrane	659
BPSS1818	**46±4 (0.0451)**	165±48 (0.2810)	85±14 (0.5401)	150±87 (0.6087)	114±16 (0.4997)	**16±1 (<0.0001)**	Hypothetical protein	Cytoplasmic membrane	638
BPSS1860	**10±2 (0.0084)**	110±20 (0.7415)	79±8 (0.3475)	**200±25 (0.0474)**	82±13 (0.3657)	**23±2 (0.0001)**	Hypothetical protein	Extracellular	316

[1] Values represent mean percent of wildtype *Bp* 1026b attachment, invasion, or intracellular replication at specified times plus or minus the standard error mean.
[2] Unpaired *t* test (two-sided) comparing each mutant to wildtype *Bp* 1026b. Significant values are bolded.
[3] As reported from *Burkholderia*.com on 09/29/2020

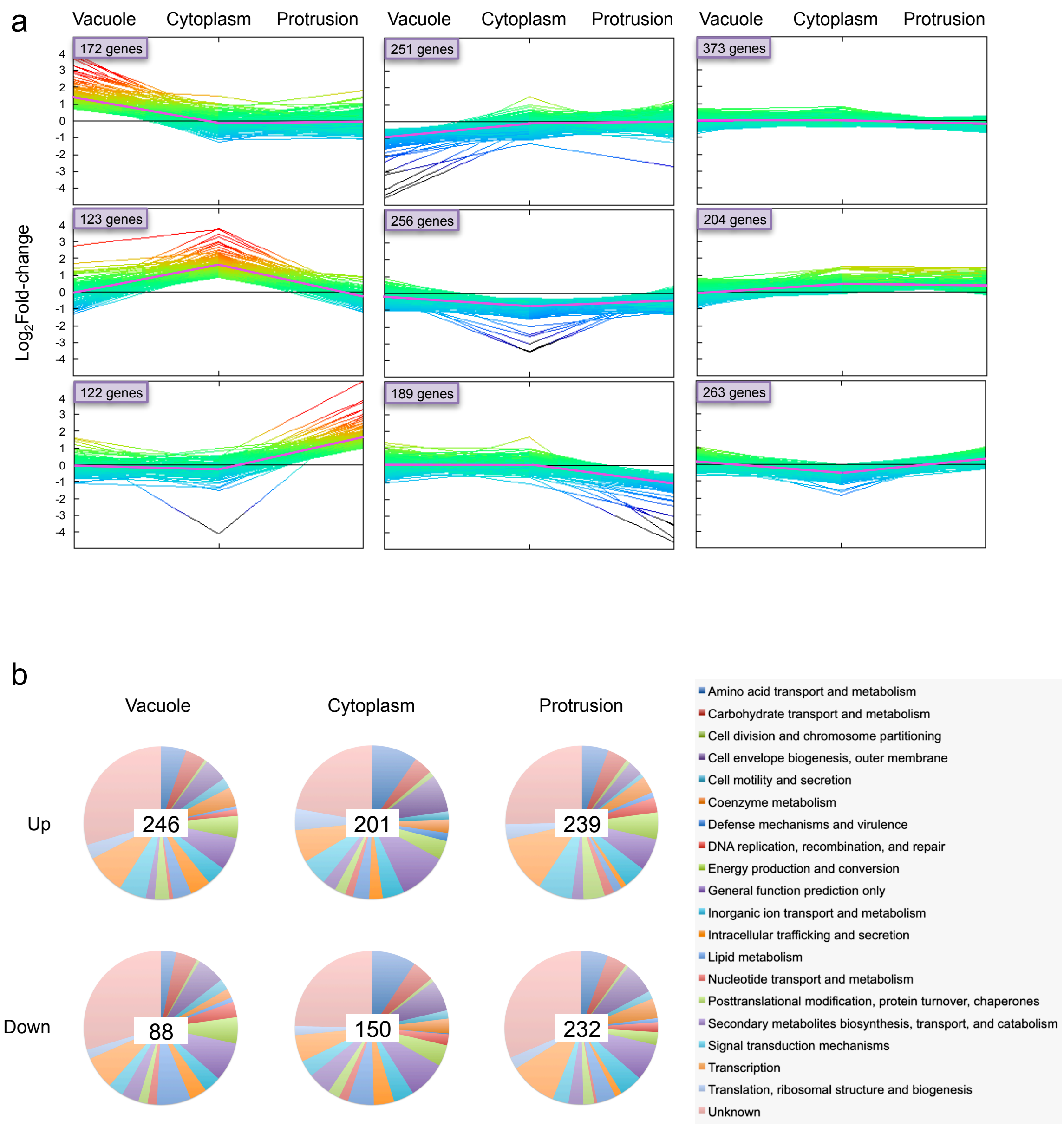

Supplementary Figure 1| Summary of the differentially expressed genes. a) Differentially expressed genes grouped into different clusters based on their expression patterns, where each line represents one gene with log_2FC represented in a rainbow color scheme (red lines are up-regulated, black lines are down-regulated). The clusters of genes that were significantly up- or down-regulated at three different infection stages were shown in the left or middle panels, respectively. The clusters of genes that remained relatively unchanged between the different intracellular infection stages and the control

condition were displayed in the right panels. The pink line indicates the average expression profile of each cluster. Number of genes in each cluster is indicated in the box at the upper left corner. b) Relative abundance of functional categories of genes differentially expressed at various stages of infection. The unknown gene (i.e., genes with neither known functions nor functional predictions) category takes up a significant portion among these functional categories, indicating that *Bp* genes possibly involved in intracellular pathogenesis are largely understudied. Total number of genes in each group is indicated in the center of the pie chart.

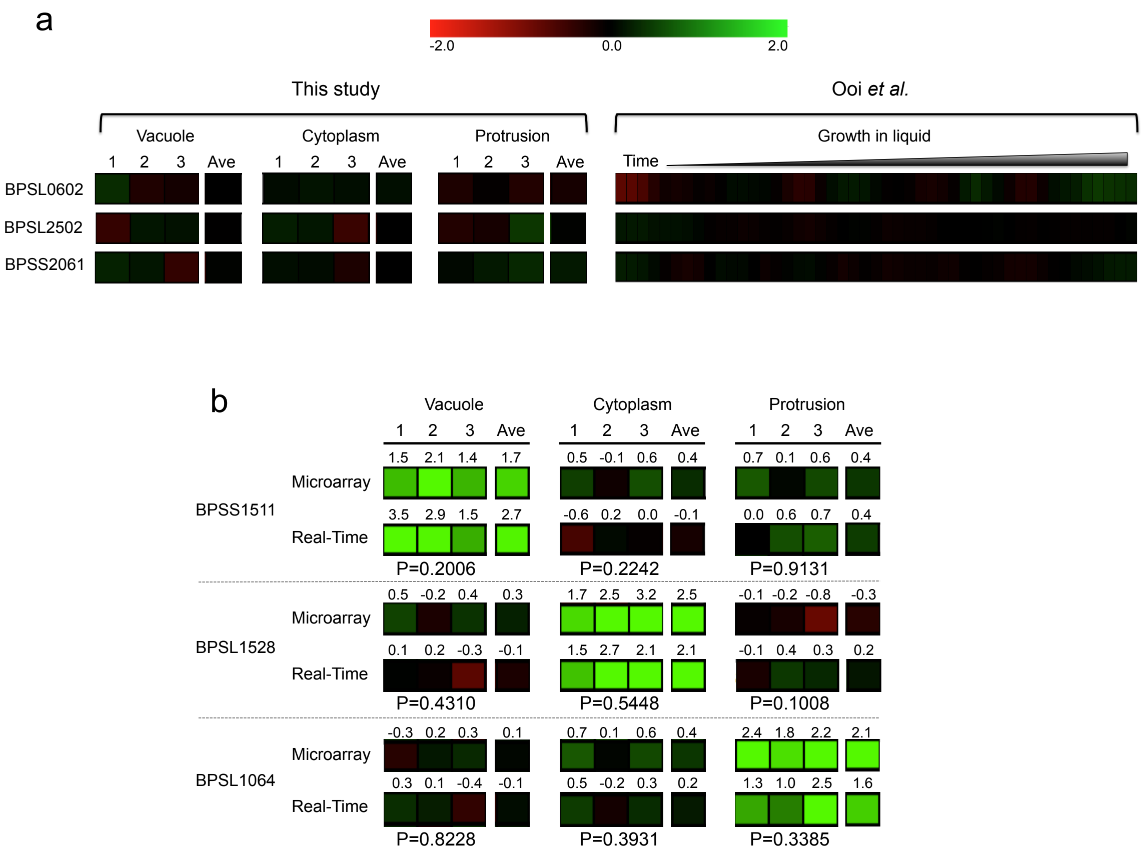

Supplementary Figure 2| Single-cell Real-Time RT-PCR validated microarray data. a) Three housekeeping genes, *BPSL0602*, *BPSL2502*, and *BPSS2061*, were chosen based on consistent expression throughout the TRANSITome data in this study, as well as previous published expression data (Ooi, *et al.,* 2013). All three housekeeping genes showed unchanged expression in the vacuole, cytoplasm, protrusion, and growth in liquid (Ooi, *et al.*). b) *BPSS1511*, *BPSL1528*, and *BPSL1064* are differentially expressed in the vacuole, cytoplasm, and protrusion, respectively, as shown in a heat map format with red-black-green color gradient as indicated in (a). Real-time RT-PCR data were obtained from three single *Bp* cells and $\log_2$fold-changes were displayed above the heat map along with averages. Real-Time RT-PCR results strongly correlated with microarray data, further validating this as a novel approach for the study of intracellular pathogens. Statistical differences between $\log_2$fold-changes of

microarray and real-time RT-PCR were calculated via two-sided unpaired t-test and P-values are presented below relevant comparisons. Scale bar for heat maps represent log_2 fold-change.

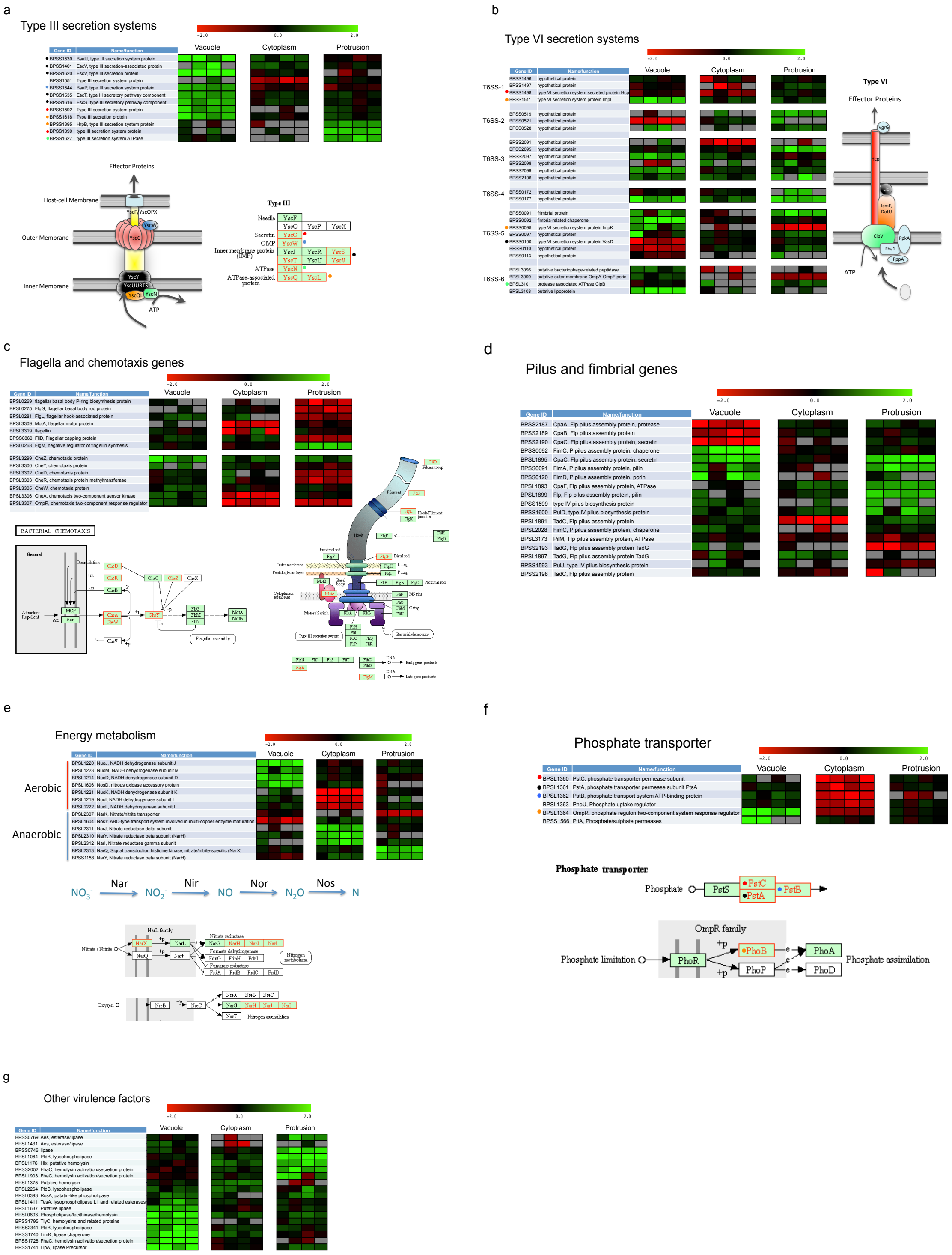

Supplementary Figure 3| Infection stage-specific expression of genes/pathways involved in (a) type III secretion systems; (b) type VI secretion systems; (c) flagellar and chemotaxis; (d) pilus and fimbriae production; (e) energy metabolism; (f) phosphate transport; and (g) other virulence factors described in

Bp. Genes with known functions that are differentially regulated are highlighted in orange boxes in the KEGG pathways. For all heat maps, the first three boxes of each infection stage represent three biological replicates, the fourth represents the mean. Scale bars for heat maps represent log_2 fold-change. All KEGG pathway maps were included with permission from Kanehisa Laboratories.

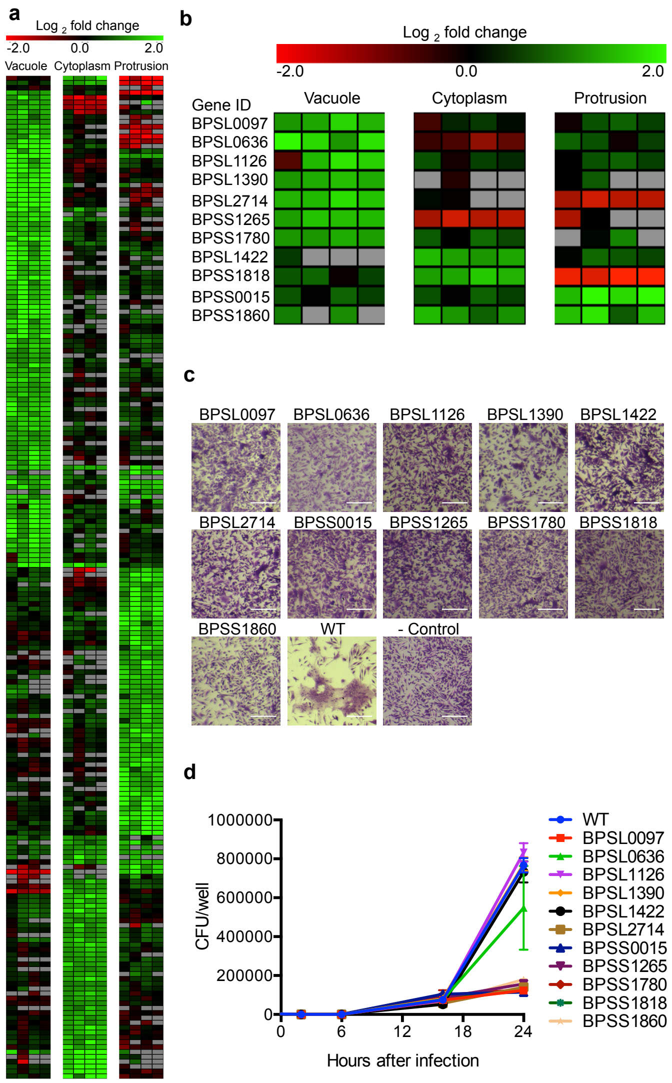

Supplementary Figure 4| TRANSITome reveals stage dependent expression of hypothetical proteins. a) Differential regulation of 206 hypothetical proteins expressed in a stage-specific manner. a) and b) First three boxes of each infection stage represent three biological replicates, the fourth represents the mean. We successfully created 191 mutants of the 206 hypothetical protein encoding genes (including several operons); mutations in five genes (BPSL0737-no homolog in *Bp* 1026b, BPSS0425, BPSS0752, BPSS1694, BPSS2179) were not successful. We screened the 191 mutants for attenuation in RAW264.7 cell infection models (i.e. MNGC formation and intracellular replication assay). b) and c) Eleven of the 191 mutants showed major defects in MNGC formation in RAW264.7 cells compared to wildtype (WT)

Bp infection. White scale bars = 100 µm. d) Majority of the eleven mutants also showed defects during intracellular replication in RAW264.7 cells and no apparent defects in liquid medium (Fig. S6a). Data represents means ± s.e.m.

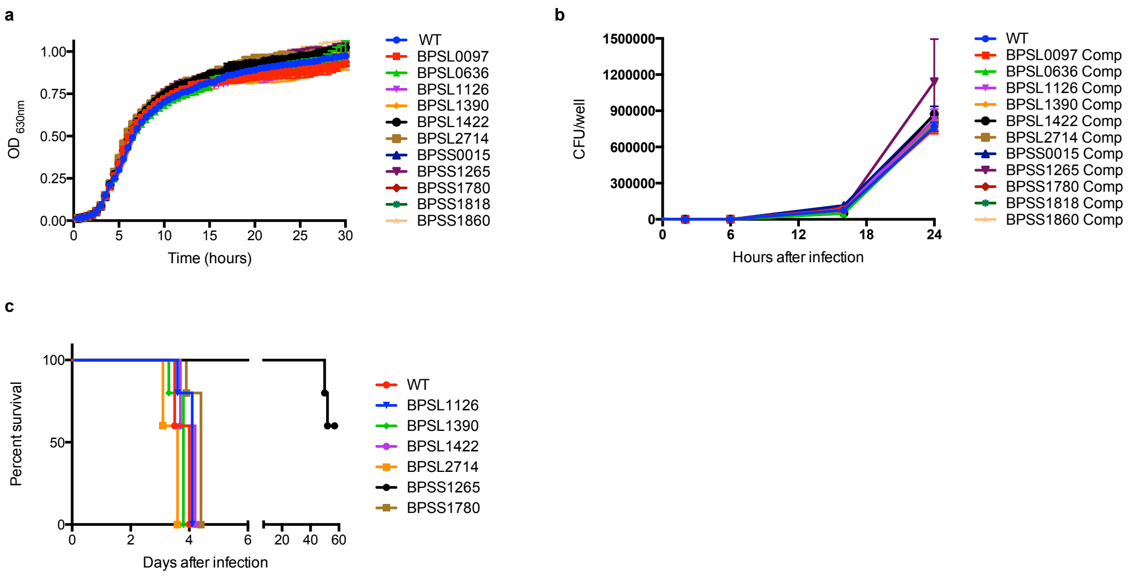

Supplementary Figure 5| Growth analysis, complementation, and *in vivo* analysis. a) Growth curves showing no growth defect of the 11 mutant strains compared to wildtype *Bp,* indicating that the defects seen during intracellular infection are not due to a reduction in mutant fitness *in vitro*. Data represents means ± s.e.m. b) Complements of the 11 mutant strains compared to wildtype *Bp* showing similar intracellular infection capabilities. No statistical differences were identified when compared to wildtype via two-sided unpaired t-test (P-value range from 0.7846-0.9972). Data represents means ± s.e.m. c) Survival curve of BALB/c mice (n=5 per infection group) infected with 4,500 CFU of each individual mutant; BALB/c mice data from only 6 of the 11 attenuated mutants are shown here, and the rest are shown and discussed elsewhere in this manuscript.

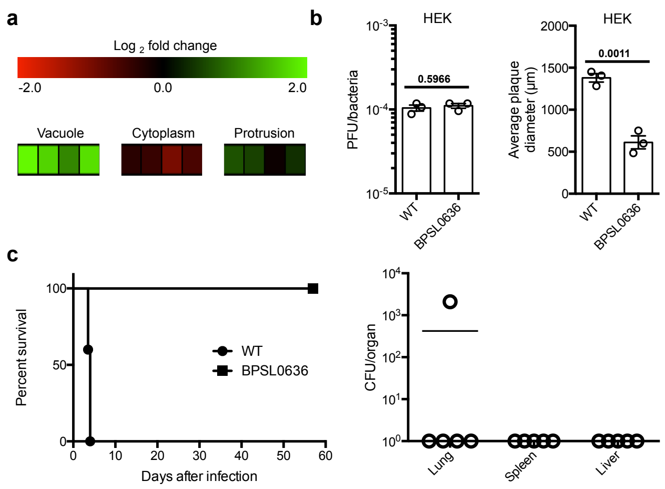

Supplementary Figure 6| Characterization of *Bp* mutant *BPSL0636*. a) *BPSL0636* is highly expressed within the macrophage vacuole. First three boxes of each infection stage represent three biological replicates, the fourth represents the mean. b) Equal numbers of plaques are formed (PFU) by wildtype *Bp* and the *BPSL0636* mutant, however, reduced plaque diameters were observed (n=3). Data represents means ± s.e.m and analyzed via two-sided unpaired t-test. *P*-values presented above relevant comparisons. c) The BPSL0636 mutant is highly attenuated in BALB/c mice (n=5) when infected intranasally with a lethal dose (4,500 CFU). Low level of *BPSL0636* mutant was recovered in lungs of only one of the infected mice, indicating the mutant was partially cleared from the lung and was unable to disseminate to other organs.
