# Supplementary material for: The Burkholderia pseudomallei intracellular ‘TRANSITome’
Source: Nat Commun. 2021 Mar 26;12:1907. doi: 10.1038/s41467-021-22169-1 (PMC7998038; doi:10.1038/s41467-021-22169-1)
Supplement: Supplementary file 2 — Description of Additional Supplementary Files [file 41467_2021_22169_MOESM2_ESM.docx]

*Description of Additional Supplementary Files*

**Title: Supplementary Data Set 1**

Description: Heat maps of all genes significantly and differentially expressed (fold-change above 2, *P* ≤ 0.05 by one-way ANOVA with multiple comparisons) in three stages of intracellular infection. Heat map is presented in a green-black-red color gradient; green color indicates up-regulation and red color indicates down-regulation, when the gene expression in each infection stage was compared to the control condition. First three boxes of each infection stage represent three biological replicates, the fourth represents the mean. Genes were sorted according to gene ID. References for known/characterized virulence factors in *Bp* are included. The first three boxes in each heat map represent independent biological replicates and the forth box is the mean.

**Title: Supplementary Data Set 2**

Description: All oligos used to generate knockout strains, complemented strains, and RT-PCR.

Title: Supplementary Movie 1

Description: An example of laser cutting and catapult-isolation (center panel) of a single Bp cell from an infected RAW264.7 macrophage cell during the protrusion stage, with fluorescent images of the same field of view before (left panel) and after (right panel) microdissection.

Title: Supplementary Movie 2

Description: Time-lapse of bright field microscopy showing MNGC formation of infected RAW264.7 cells. Left panel is RAW264.7 cells infected by wildtype Bp; right panel shows RAW cells infected by BPSL0636 mutant. RAW cells infected by BPSL0636 mutant exhibited significantly delayed MNGC formation. One frame was taken every 5 minutes for 24 hours.

Title: Supplementary Movie 3

Description: Time-lapse of bright field microscopy showing wildtype Bp attachment to RAW264.7 cells. Individual Bp cells were traced in the identical videos using ImageJ (left video untraced and right video shows traced wildtype Bp bacteria encountering host cells).

Title: Supplementary Movie 4

Description: Time-lapse of bright field microscopy showing the BPSL0097 mutant unable to attach to RAW264.7 cells. Individual Bp cells were traced in the identical videos using ImageJ (left video untraced and right video shows traced BPSL0097 mutant bacteria encountering host cells).

Title: Supplementary Movie 5

Description: Time-lapse of bright field microscopy showing the BPSS1860 mutant unable to attach to RAW264.7 cells. Individual Bp cells were traced in the identical videos using ImageJ (left video untraced and right video shows traced BPSS1860 mutant bacteria encountering host cells).

Title: Supplementary Movie 6

Description: Time-lapse of bright field microscopy showing MNGC formation of infected RAW264.7 cells. Left panel shows RAW264.7 cells infected by wildtype Bp; right panel shows RAW264.7 cells infected by the BPSS1818 mutant. RAW264.7 cells infected by the BPSS1818 mutant exhibited MNGC formation compared to wildtype Bp, but the cytoskeleton of the MNGC infected with the BPSS1818 mutant appeared to be extended as compared to wildtype Bp infected cells. At the end of infection, BPSS1818 infected cells showed “stretched out” phenotype. One frame was taken every 5 minutes for 24 hours.
