# Supplementary material for: The Burkholderia pseudomallei intracellular ‘TRANSITome’
Source: Nat Commun. 2021 Mar 26;12:1907. doi: 10.1038/s41467-021-22169-1 (PMC7998038; doi:10.1038/s41467-021-22169-1)
Supplement: Supplementary file 11 — Reporting Summary [file 41467_2021_22169_MOESM11_ESM.pdf]

nature research

Corresponding author(s): Tung T. Hoang

Last updated by author(s): Feb 8, 2021

Reporting Summary

Nature Research wishes to improve the reproducibility of the work that we publish. This form provides structure for consistency and transparency in reporting. For further information on Nature Research policies, see our Editorial Policies and the Editorial Policy Checklist.

Statistics

For all statistical analyses, confirm that the following items are present in the figure legend, table legend, main text, or Methods section.

n/a	Confirmed	
☐	☒	The exact sample size (n) for each experimental group/condition, given as a discrete number and unit of measurement
☐	☒	A statement on whether measurements were taken from distinct samples or whether the same sample was measured repeatedly
☐	☒	The statistical test(s) used AND whether they are one- or two-sided<br>*Only common tests should be described solely by name; describe more complex techniques in the Methods section.*
☐	☒	A description of all covariates tested
☒	☐	A description of any assumptions or corrections, such as tests of normality and adjustment for multiple comparisons
☐	☒	A full description of the statistical parameters including central tendency (e.g. means) or other basic estimates (e.g. regression coefficient) AND variation (e.g. standard deviation) or associated estimates of uncertainty (e.g. confidence intervals)
☐	☒	For null hypothesis testing, the test statistic (e.g. F, t, r) with confidence intervals, effect sizes, degrees of freedom and P value noted<br>*Give P values as exact values whenever suitable.*
☒	☐	For Bayesian analysis, information on the choice of priors and Markov chain Monte Carlo settings
☒	☐	For hierarchical and complex designs, identification of the appropriate level for tests and full reporting of outcomes
☒	☐	Estimates of effect sizes (e.g. Cohen's d, Pearson's r), indicating how they were calculated

Our web collection on statistics for biologists contains articles on many of the points above.

Software and code

Policy information about availability of computer code

Data collection	GenePix Pro software 5.1, AxioVision 4.9.1, Image Lab 2.0.1, and Gen5 2.06
Data analysis	TIGR_Spotfinder 3.2.1, MIDAS 2.21, Mev 4.5.1, ImageJ 1.49, and Prism 6.0

For manuscripts utilizing custom algorithms or software that are central to the research but not yet described in published literature, software must be made available to editors and reviewers. We strongly encourage code deposition in a community repository (e.g. GitHub). See the Nature Research guidelines for submitting code & software for further information.

Data

Policy information about availability of data

All manuscripts must include a data availability statement. This statement should provide the following information, where applicable:

- Accession codes, unique identifiers, or web links for publicly available datasets
- A list of figures that have associated raw data
- A description of any restrictions on data availability

The microarray datasets generated in this study have been deposited in NCBI's Gene Expression Omnibus and are accessible through GEO Series accession number GSE156938.
Gene description, function prediction, and functional category assignment was assisted for some genes using Burkholderia Genome database (http://www.burkholderia.com).

Field-specific reporting

Please select the one below that is the best fit for your research. If you are not sure, read the appropriate sections before making your selection.

☒ Life sciences ☐ Behavioural & social sciences ☐ Ecological, evolutionary & environmental sciences

For a reference copy of the document with all sections, see nature.com/documents/nr-reporting-summary-flat.pdf

Life sciences study design

All studies must disclose on these points even when the disclosure is negative.

Sample size	No sample size calculation was performed, sample sizes were chosen according to the standard practices in the field. At least three replicates were done for each condition, which is sufficient for statistical analysis.<br>Three biological replicates (individual Burkholderia pseudomallei cells from each of the host cell infection stages) are isolated and used for transcriptomic analysis.<br>All in vitro characterization of the mutant strains (i.e. attachment assay, intracellular replication assay, plaque assay, growth study, fluorescence microscopy, and TEM) were performed in triplicate, unless otherwise indicated.<br>Groups of 5 animals were used for the infection study.
Data exclusions	No data was excluded.
Replication	All experiments were done in at least duplicate, and replications were successful.
Randomization	Initial screen of the 191 mutants were performed in a blinded and random fashion to identify mutant with defects in virulence in macrophage cells.<br>Microscopy fields for imaging and measurement were randomly selected.<br>Animal purchased for the infection study were randomly allocated to different challenge groups, according to their grouping upon arrival.
Blinding	For the initial screen of the 191 mutants, investigators were blinded to the names of the mutants until defects were observed.<br>For animal study, investigators were not blinded to the strains used.

Reporting for specific materials, systems and methods

We require information from authors about some types of materials, experimental systems and methods used in many studies. Here, indicate whether each material, system or method listed is relevant to your study. If you are not sure if a list item applies to your research, read the appropriate section before selecting a response.

Materials & experimental systems

n/a	Involved in the study	
☐	☒	Antibodies
☐	☒	Eukaryotic cell lines
☒	☐	Palaeontology and archaeology
☐	☒	Animals and other organisms
☐	☒	Human research participants
☒	☐	Clinical data
☒	☐	Dual use research of concern

Methods

n/a	Involved in the study	
☒	☐	ChIP-seq
☒	☐	Flow cytometry
☒	☐	MRI-based neuroimaging

Antibodies

Antibodies used	Mouse anti-HA antibody conjugated with Alexa Fluor® 594, ThermoFisher Scientific, Cat# A-21288, Lot# 1740027.<br>Goat-anti-mouse antibody with Alexa Fluor® 488 and 10 nm colloidal gold conjugate,ThermoFisher Scientific, Cat# A-31561, Lot# 1348715.<br>β-tubulin antibody conjugated with Alexa Fluor 594, Cell Signaling Technology, Cat# 7634S, Lot# 1.<br>Goat anti-human Ig-HRP antibody, Invitrogen, Cat# AHI0704, Lot# 1228096.
Validation	Antibodies used in this study are all purchased from commercial sources, their specificities and sensitivities are noted on commercial product info pages at the locations specified below:<br>Mouse anti-HA antibody conjugated with Alexa Fluor® 594: https://www.thermofisher.com/antibody/product/HA-Tag-Antibody-clone-16B12-Monoclonal/A-21288.<br>Goat-anti-mouse antibody with Alexa Fluor® 488 and 10 nm colloidal gold conjugate: https://www.thermofisher.com/antibody/product/Goat-anti-Mouse-IgG-H-L-Secondary-Antibody-Polyclonal/A-31561.<br>β-tubulin antibody conjugated with Alexa Fluor 594: https://www.cellsignal.com/products/antibody-conjugates/b-tubulin-9f3-rabbit-mab-alexa-fluor-594-conjugate/7634.

Goat anti-human Ig-HRP antibody: http://tools.thermofisher.com/content/sfs/manuals/AHI0704_Lot1228096_gt_x_hu_IgG_HRP_man.pdf.

All antibodies have been confirmed in this study for their specificities using appropriate negative and positive controls done in triplicate.

Eukaryotic cell lines

Policy information about cell lines

Cell line source(s)	Murine macrophage cell line RAW264.7, human embryonic kidney cell line HEK293T, and human neuroblastoma cell line HTB-11 a.k.a. SK-N-SH cells are in-house collections of widely used and commercially available cell lines, originally obtained from ATCC.
Authentication	None of the cell lines used were authenticated.
Mycoplasma contamination	Cell lines were not tested for Mycoplasma contamination.
Commonly misidentified lines (See ICLAC register)	None of the cell lines were commonly misidentified lines.

Animals and other organisms

Policy information about studies involving animals; ARRIVE guidelines recommended for reporting animal research

Laboratory animals	BALB/c mice of either sex between 4 and 6 weeks of age were purchased from Charles River Laboratory.
Wild animals	No wild animals were used in this study.
Field-collected samples	No field-collected samples were used in this study.
Ethics oversight	The animal studies described in this manuscript were conducted in compliance with the NIH (National Institutes of Health) Guide for the Care and Use of Laboratory Animals and approved by the Institutional Animal Care and Use Committee at the University of Hawaii at Manoa (Protocol No. 10-1073).

Note that full information on the approval of the study protocol must also be provided in the manuscript.

Human research participants

Policy information about studies involving human research participants

Population characteristics	Blood samples were taken from confirmed melioidosis patients.
Recruitment	All participation was voluntary and samples were collected following informed consent.
Ethics oversight	Ethics approval provided by Townsville Hospital and Health Services, Australia (HREC/12/QTHS/213)

Note that full information on the approval of the study protocol must also be provided in the manuscript.
